# Intravacuolar Pathogens Hijack Host Extracellular Vesicle Biogenesis to Secrete Virulence Factors

**DOI:** 10.3389/fimmu.2021.662944

**Published:** 2021-04-20

**Authors:** Anna Gioseffi, Mariola J. Edelmann, Peter E. Kima

**Affiliations:** Department of Microbiology and Cell Science, Institute of Food and Agricultural Sciences, University of Florida, Gainesville, FL, United States

**Keywords:** extracellular vesicles, exosomes, infections, Leishmania, Mycobacterium, Plasmodium, Toxoplasma

## Abstract

Extracellular vesicles (EVs) have garnered significant interest in recent years due to their contributions to cell-to-cell communication and disease processes. EVs are composed of a complex profile of bioactive molecules, which include lipids, nucleic acids, metabolites, and proteins. Although the biogenesis of EVs released by cells under various normal and abnormal conditions has been well-studied, there is incomplete knowledge about how infection influences EV biogenesis. EVs from infected cells contain specific molecules of both host and pathogen origin that may contribute to pathogenesis and the elicitation of the host immune response. Intracellular pathogens exhibit diverse lifestyles that undoubtedly dictate the mechanisms by which their molecules enter the cell’s exosome biogenesis schemes. We will discuss the current understanding of the mechanisms used during infection to traffic molecules from their vacuolar niche to host EVs by selected intravacuolar pathogens. We initially review general exosome biogenesis schemes and then discuss what is known about EV biogenesis in *Mycobacterium, Plasmodium, Toxoplasma*, and *Leishmania* infections, which are pathogens that reside within membrane delimited compartments in phagocytes at some time in their life cycle within mammalian hosts. The review includes discussion of the need for further studies into the biogenesis of EVs to better understand the contributions of these vesicles to host-pathogen interactions, and to uncover potential therapeutic targets to control these pathogens.

## Introduction

There are myriad facets of host–pathogen interactions that contribute to infection outcomes. Such is the case with intracellular pathogenesis, which includes organisms that use the host biosynthetic machinery to propagate themselves, organisms that reside in the cell cytoplasm and organisms that reside within vacuolar compartments in host cells. Progress in our understanding of mechanisms deployed by pathogens to exploit or subvert host mechanisms is uneven. It should therefore be enlightening to consider current knowledge of pathogen-host interactions by pathogens that share important characteristics. Our focus will be on pathogens that reside within membrane delimited vacuolar compartments in the cell. Even among this relatively small subset of pathogens, there is great diversity in pathogen strategies to interface with the cell, including differences in the composition and interactions of the pathogen-containing vacuolar compartments. Each pathogen elaborates unique strategies to translocate molecules from their intravacuolar niche into the cell cytosol or to other host cell organelles where they target host cell processes. It is presumed that some of the molecules that are released into the cell cytosol may access the exosome biogenesis machinery of host cells. The intravacuolar pathogens that we will discuss infect phagocytes at some point in their life cycle. Phagocytes are residents of tissues where they play important roles in tissue homeostasis and disease [reviewed in (1)]. Phagocytes are also recruited to sites of tissue damage from infection or other insults, where they proceed to limit the infection by various strategies, including ingesting and destroying any intruders, elaboration of molecular mediators to recruit and activate cells, removal of damaged host cells, or attempts to wall off the site. Paradoxically, phagocytes are unwitting hosts of a wide range of pathogens, including viruses, bacteria, and eukaryotic parasites. Infection of phagocytes and other host cells by the intravacuolar pathogens discussed here results in diseases that cause tremendous human suffering.

Numerous studies have shown that extracellular vesicles (EVs) released by phagocytic cells infected with intracellular pathogens hold infection-specific molecular cargo that may contribute to pathogenesis and host immune responses [reviewed in (2–4)]. Despite the abundance of proteomic and functional data, the process of EV biogenesis during infection and mechanisms by which pathogen-derived molecules are packaged into host exosomes are poorly understood. Insightful studies of EV biogenesis in the context of infection have focused on viral pathogens, revealing that many viruses utilize the endosomal sorting complex required for transport (ESCRT) machinery for viral egress. However, the biogenesis of EVs from infection by intracellular bacteria or intracellular eukaryotic pathogens remains poorly understood. The purpose of this review is to discuss the current understanding of the mechanisms of EV biogenesis in the context of intravacuolar pathogen infection to highlight each pathogen’s strategies to exploit host EV biogenesis schemes. We initially discuss universal characteristics and EV biogenesis schemes, after which the situation in the context of infection is considered.

## Exosome Composition and Biogenesis

Extracellular vesicles are secreted by all mammalian cells and can be isolated from various bodily fluids, including blood, urine, breast milk, tears, and cerebrospinal fluid (5). Extracellular vesicles have garnered significant interest in recent years because of their ability to transfer potentially important intercellular communication mediators, including proteins (6). This function is of particular interest in the context of host-pathogen interactions, as EVs may be critical mediators of host-pathogen communication and contribute to pathogenesis. Exosomes may be classified by their size, cell of origin, biogenesis, or proposed function (7–9). Apoptotic bodies are membrane-enveloped vesicles that range in diameter between 50 - 5000nm released *via* the blebbing of cells undergoing apoptosis (5, 8, 10). The existence of apoptotic bodies has been long known and studied. Healthy cells also shed extracellular vesicles, including microvesicles and exosomes (11). Microvesicles are membrane-bound vesicles released through outward budding and fission of the plasma (11, 12). Microvesicles are a heterogeneous group of vesicles that have been referred to by various terms including, ectosomes, shedding vesicles, microparticles, or platelet dust; that range in size from 100 - >1000nm in diameter (12, 13). Lastly, exosomes are membrane-enclosed vesicles between 50-150 nm in diameter that are secreted when multivesicular bodies (MVBs) in the endosomal pathway fuse with the plasma membrane and release intraluminal vesicles (ILVs) into the extracellular space (13). Together, these three classes of vesicles whose minimal characteristics and nomenclature have been standardized (14), are set apart in significant ways by both their biogenesis and biological functions.

Exosomes were first described in 1987 (15) and have since been found to be secreted by nearly all eukaryotic cells except for mature red blood cells, which do not possess endocytic capacities (16). Exosomes are secreted by healthy cells continuously, and their cargo and pattern of release can be altered by conditions of stress or infection (17). These nanosized vesicles contain thousands of cell-specific molecules, including proteins, nucleic acids, and lipids, enclosed within a single lipid bilayer membrane (18). Some of these molecules are conserved across exosomes from different origins, including tetraspanins (CD9, CD63, and CD81), proteins involved in intracellular trafficking (Rab GTPases, annexins), chaperones (Hsc70), biogenesis factors (ALIX), and proteins associated with signal transduction (14-3-3 proteins) (11). However, the exact composition of exosomes is dynamic and reflective of cellular context and the health of its cell of origin. Because the composition of exosomes is adaptive and reflective of the cell environment and condition, they are intriguing candidates for disease biomarker discovery through the isolation and screening of exosomes from patient bodily fluids (19).

Exosomes are of endosomal origin and are created when intraluminal vesicles (ILVs) form by inward budding of the early endosome (EE) (20). Early endosomes containing ILVs then mature into MVBs and are directed either to the lysosome for degradation or fuse with the plasma membrane to release the ILVs to the extracellular space as exosomes (20). Exosomes contain an assortment of molecular cargo, including membrane proteins with exposed extracellular domains on the exosomal surface and cytosolic proteins enclosed within their lumen (21). The protein composition of exosomes is specific and not reflective of the total cell, indicating that there are specific mechanisms to control the loading of select molecules into exosomes (22). Exosome biogenesis and the process of selective protein loading remain poorly understood phenomena. It is known, however, that the process of exosome biogenesis is driven by at least two general mechanisms: endosomal sorting complexes required for transport (ESCRT)-dependent and ESCRT-independent (tetraspanin or lipid-dependent) processes (22).

### ESCRT-Dependent Pathway

The ESCRT machinery was first shown to play a role in sorting proteins into MVBs in yeast in 2001 (23). The ESCRT complex comprises nearly 30 proteins assembled into five coordinating subcomplexes that function in a stepwise fashion (24): ESCRT-0, -I, -II, and -III, and associated AAA ATPase Vps4 complex (25). The primary function of ESCRT in ancestral organisms was to constrict and sever narrow membrane necks by a currently unknown mechanism of membrane scission (26). The ESCRT machinery is involved in many eukaryotic cellular processes, including the sorting of ubiquitinated proteins into ILVs for lysosomal degradation, viral egress, and membrane scission during cytokinesis (27). More recently, ESCRT components such as Tsg101 and Alix have been identified in exosome preparations from different sources, which suggested the involvement of ESCRT in the biogenesis of these vesicles (25).

The ESCRT subcomplexes perform a series of synchronized tasks to drive both cargo loading and physical membrane-remodeling and scission, leading to the production of ILVs: ESCRT-0 sequesters ubiquitinated cargo, ESCRT-I, -II, and -III control ILV budding, and Vps4 regulates membrane scission (28). ESCRT-0 consists of two subunits, hepatocyte growth factor-regulated tyrosine kinase substrate (Hrs) and signal-transducing adaptor molecule 1/2 (STAM1/2), and can bind both phosphatidylinositol 3-phosphate (PtdIns3P) and ubiquitin, providing membrane recruitment, endosomal specificity, and interaction with ubiquitinated target proteins (29). ESCRT-0, ESCRT-I, and ESCRT-II all contain ubiquitin-binding domains, suggesting that ubiquitination is important for the selective loading of cargo proteins into exosomes (30). ESCRT-I consists of tumor susceptibility gene 101 (Tsg101), Vps28, Vps37, and multivesicular body 12 (hMvb12) and interacts with both ESCRT-0 and ESCRT-II using domains located on opposite ends of the complex (31). ESCRT-I then recruits ESCRT-II, a Y-shaped subcomplex consisting of EAP30, EAP45, and two subunits of EAP20, which cooperates with ESCRT-0 to provide further endosomal specificity and recruits ESCRT-III (32). ESCRT-III is composed of four main subunits- charged multivesicular body proteins (CHMPs) CHMP2A, B, CHMP6, CHMP3, and CHMP4A,B,C- and several adaptors and accessory proteins such as ALIX, which can recruit a deubiquitinating enzyme and is essential for cargo loading (33, 34), Deubiquitylation appears to be involved in loading cargo into ILVs, which are destined for degradation, while exosomal proteins remain ubiquitinated (25). The multimeric mechanoenzyme class I AAA (ATPase associated with various cellular activities) ATPase Vps4 is required to remove assembled ESCRT-III from the membrane before vesicle formation (35).

The PDZ (postsynaptic density protein, disc large, and zonula occludens) domain-containing protein syntenin is important for the biogenesis of a specific subclass of CD63-positive ILVs. Syntenin interacts with ALIX, pieces of the ESCRT machinery, and membrane receptors such as syndecans to produce ILVs using an alternative ESCRT pathway of cargo recruitment and vesicle budding (36), suggesting that ESCRT-dependent exosome production may be a flexible process resulting in a heterogeneous population of vesicles (9).

### ESCRT-Independent Pathway

Depletion of all four ESCRT subcomplexes is insufficient to block exosome secretion entirely in eukaryotic cell lines but does result in drastic changes in cell morphology, such as enlarged, empty MVBs and irregularly shaped ILVs (37). Therefore, these larger, heterogeneous vesicles are produced by ESCRT-independent mechanisms that rely on other lipids and proteins for the loading and budding of exosomes (25).

Lipidomic analysis of EVs reveals that exosomes contain an overall low concentration of lipids, resulting in a high protein/lipid ratio (38). The lipid composition of exosomes is reminiscent of detergent-resistant lipid rafts, and it is enriched in cholesterol, ceramide, and sphingolipids essential for the ESCRT-independent sorting of cargo into ILVs (39). Inhibition of ceramide production using the neutral sphingomyelinase (nSMase) inhibitor GW4869 showed a marked reduction in exosome release, which appeared to be specific and independent from ESCRT-dependent mechanisms rather than a derangement of the entire endosomal system (39). Furthermore, ILVs destined to be secreted as exosomes contain ceramide while ILVs intended for lysosomal degradation contain another related lipid, lysobisphosphatidic acid (LBPA), which is absent in exosomes, suggesting that lipid composition is involved in the determination of vesicle fate (40, 41).

**Figure 1:**
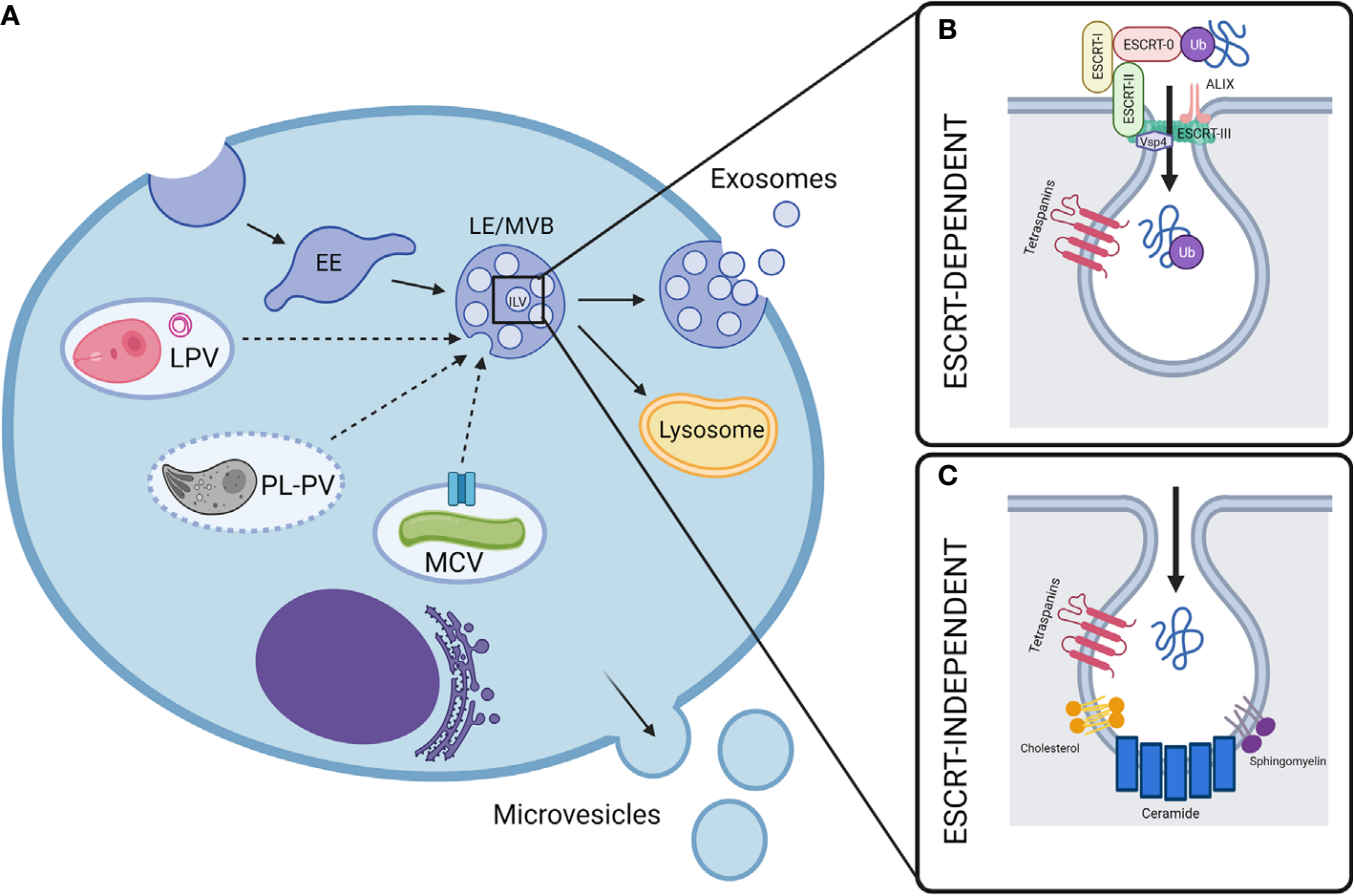
Biogenesis of extracellular vesicles in intravacuolar pathogen infections. **(A)** Mammalian cell-derived extracellular vesicles include exosomes and microvesicles, which can be characterized by their biogenesis, size, and composition. While microvesicles are generated by the direct outward budding of the cell’s outer membrane, exosomes are derived from an endocytic origin. First, early endosomes (EE) undergo inward budding. This forms intralumenal vesicles (ILVs) inside what is then called the late endosomal vesicle (LE) or multivesicular body (MVB). Depending on molecular signals, the MVB may then be destined for degradation by fusing with lysosomes or will fuse with the plasma membrane, releasing the ILVs to the extracellular space where they are then called exosomes. In the context of intravacuolar pathogens, exosomes are a possible mechanism for the release of pathogen molecules from infected cells. The exact mechanisms for how these pathogen molecules escape their respective vacuoles and are trafficked to host exosomes is not fully understood, but it is likely to be unique for each pathogen as discussed in this review. The composition of pathogen-containing vacuoles is diverse and requires unique methods for the exchange of molecules between host and pathogen. *Mycobacteria* utilize secA, type VII secretion system (represented by blue channel), and possibly other secretion systems to secrete exosome-bound proteins from the *Mycobacterium* containing vacuole (MCV). *Plasmodium* containing vacuoles (PL-PVs) conversely contain pores (represented by dashed vacuole boarder) through which small molecules may pass freely between the PV lumen and host cytosol, presenting easy access of pathogen molecules to host cytosol and exosome processes. *Leishmania* parasitophorous vacuoles (LPVs) are dynamic compartments that interact with the host’s endocytic and secretory pathways. Multimembranous structures within LPVs whose cellular origin is not known, may transport molecules from LPVs to host cell organelles including MVBs. Dashed arrows represent speculative pathways for trafficking of pathogen molecules into host ILVs. **(B, C)** Exosome biogenesis can occur by two general mechanisms- either ESCRT-dependent or ESCRT-independent. **(B)** ESCRT, or endosomal sorting complexes required for transport, is a specialized multi-subunit complex which allows for the recruitment of ubiquitinated proteins and the inward budding and scission of ILVs. ESCRT-0 recruits ubiquitinated proteins while ESCRT-I, ESCRT-II, and ESCRT-III facilitate ILV budding, and finally Vsp4 facilitates membrane scission. Accessory proteins such as ALIX are also involved which perform deubiquitylation for cargo loading. **(C)** Less is currently known about the mechanisms of ESCRT-independent exosome biogenesis, however it is proposed that ceramide and other lipids, such as sphingomyelin and cholesterol, as well as tetraspanins play a part in the trafficking of proteins and inward budding. Created with BioRender.com.

However, depletion of ceramide has no effect on MVB biogenesis or exosome secretion in human melanoma cells (42). In these cells, the tetraspanin CD63 was involved in the ESCRT-independent sorting of cargo into ILVs (42). Other tetraspanins, including CD82 and CD9, have also been shown to participate in the ceramide-dependent biogenesis of exosomes in healthy primary cells (43). Tetraspanin-enriched microdomains (TEMs) may act as sorting platforms for cargo molecules during the ESCRT-independent biogenesis of ILVs (**Figure 1C**). However, the exact mechanisms by which tetraspanins facilitate exosomal cargo sorting are unknown (44).

### Cargo Sorting: Post-Translational Modification of Target Proteins

Exosomal protein composition is selective and dynamic, suggesting that there are regulated mechanisms involved in loading specific target molecules during vesicle biogenesis. Mass spectrometry analysis of exosomes reveals that post-translational modifications (PTMs) such as ubiquitination (mono-ubiquitination and poly-ubiquitination), the addition of small ubiquitin-related modifier (SUMOylation), phosphorylation, and glycosylation are all common modifications of exosome proteins and may be involved in the selective sorting of cargo molecules (45).

Ubiquitin can be bound to a target protein at numerous positions *via* the isopeptide bond of C-terminal glycine of ubiquitin with the ϵ-amino group of a lysine residue present in the target protein, resulting in a complex network of modifications (45). The relationship between ubiquitination and cargo sorting is unclear as protein sorting can also occur through ESCRT and ubiquitin- independent mechanisms (22). SUMO, another ubiquitin-like modifier, has also been shown to modify exosomal proteins and influence miRNAs packaging into exosomes (46). Also, phosphorylation has been found to work in conjunction with ubiquitination and lipid rafts to regulate the sorting of some specific subsets of proteins into the exosomes (47). Despite these observations, current studies are so far unable to unravel the complicated network of PTMs and their involvement in selective cargo trafficking and vesicle fate.

### The Potential of EVs in Infectious Disease

Extracellular vesicles are known to carry cargo whose composition is unique to the cell of origin and is modulated by the cell’s environment and growth conditions. This implies that the profile of cargo molecules in EVs from cells experiencing stressful growth conditions, including infection, will differ somewhat from the molecular profile of EVs released under non-stressful growth conditions. EVs therefore carry biomarkers that can inform on the staging of a disease process. It is no surprise therefore that exosomes are being touted as a source for promising biomarkers for cancer diagnosis and that they represent new targets for cancer therapy (48). In infectious diseases as well, knowledge of EV composition modulated by infection could similarly be exploited for development of biomarkers for diagnosis of infection and identification of immune targets. Exosomes released by macrophages infected with several intracellular pathogens, including *Salmonella enterica* and *Mycobacterium tuberculosis*, have been demonstrated to include pathogen antigens, stimulate a pro-inflammatory response in naïve cells (49) and protect against subsequent infection challenges (50), suggesting that exosomes may also be useful as cell-free vaccines against infectious pathogens.

## Intravacuolar Prokaryotic Pathogen

### Roles of Exosomes in Infections Caused by *Mycobacteria*

*Mycobacteria* are acid-fast bacteria that prefer an intracellular lifestyle. *Mycobacterium tuberculosis* is a human pathogen that causes tuberculosis, and *M. avium* complex bacteria also lead to lung infection, although primarily in immunocompromised individuals. Finally, *M. bovis* bacillus Calmette-Guérin (BCG) is a vaccine strain of *Mycobacterium*. *Mycobacteria* can infect several cell types, such as neutrophils, macrophages, and monocytes, although alveolar macrophages appear to be the preferred host of *Mycobacterium tuberculosis* in the early phases of infection (51). During the host cell infection, *Mycobacterium* binds to the macrophage surface, followed by internalization of the bacteria into *Mycobacterium* containing vacuoles (MCV) that do not fuse with lysosomes, which ensures that the bacterium survives in the vacuole (52). This bacterium recruits several host proteins to the MCV surface, including rab5, to prevent phagolysosomal fusion at the early endosome stage. This phenomenon is reflected by the fact that MCVs have a low abundance of lysosomal markers, such as CD63, LAMP-1 and -2, or rab7 (53, 54). *Mycobacterium* uses the SecA2 pathway to secrete SapM and PknG effector proteins that affect phagosome and autophagosome maturation (55).

While surviving in the MCV, *Mycobacterium* components, pathogen-associated molecular patterns (PAMPs) appear to be translocated from the vacuole *via* secretion systems. It has been shown that cell wall constituents of *M. avium*, glycopeptidolipids, are released by infected macrophages (56). These glycopeptidolipids are trafficked from the MCV to multivesicular bodies (MVBs), and the trafficking process is enabled by the endocytic network. After the MVBs fuse with the plasma membrane, the released exosomes still contain the glycopeptidolipids, which are transferred from infected to uninfected macrophages where they stimulate proinflammatory mediators *via* Toll-like receptors (TLRs) -2, -4, that are dependent on the MyD88 pathway (56). Other studies showed that exosomes isolated from the bronchoalveolar lavage fluid (BALF) of *M. bovis* BCG–infected mice also stimulated proinflammatory responses in macrophages, resulting in TNF-α production (57). These vesicles contained the *Mycobacteria* components lipoarabinomannan and the 19-kDa lipoprotein. Intranasal vaccination with exosomes isolated from macrophages previously infected with *M. bovis* BCG and *M. tuberculosis* stimulated TNF-α or IL-12 production and aided in neutrophil and macrophage recruitment to the lung of mice (57). A similar experiment was performed with exosomes isolated from *Mycobacterium*-infected macrophages, which were used to treat naïve bone marrow-derived macrophages, revealing that these exosomes also stimulate other cytokines, such as GCSF, sICAM1, IL-1ra, MIP-1a, MIP-1b, MIP-2, RANTES, and MCP-5. In addition to inducing the release of cytokines, exosomes from *Mycobacteria*-infected cells have been shown to induce migration of macrophages. This phenomenon has been demonstrated *in vivo*, based on the fact that the intranasally injected exosomes resulted in the recruitment of CD11b+ cells into the lung (58).

Apart from exosomes’ effect on innate immune cells, exosomes derived from *Mycobacteria*-infected cells also stimulate adaptive immunity in the *M. tuberculosis* model. Exosomes derived from macrophages exposed to culture filtrate proteins (CFP) of *M. tuberculosis* induced pathogen‐specific IFN‐γ and IL‐2‐expressing CD4+ and CD8+ T cells. This Th1-biased immune response was specific to mice vaccinated with exosomes obtained from CFP-treated macrophages, while the BCG vaccine boosted the Th2 response. Those exosomes were also shown to prime a protective immune response at a level comparable to BCG and provided a booster to a previous BCG immunization, leading to a decrease in mycobacterial count in lung and spleen of mice challenged with aerosolized *M. tuberculosis* (50). Exosomes released *in vivo* during infection with *M. tuberculosis* also contribute to T cell response, which was shown by using rab27a-deficient mice (59), which have a defect in the exosome generation (60). The deletion of rab27a was correlated with an increased bacterial burden and decreased T cell activation, indicating the importance of exosomes in the T cell function (59).

### Trafficking of Mycobacterial Proteins to Exosomes

Mycobacterial proteins are trafficked to exosomes, shown first for the 19-kDa lipoprotein (57). A proteomic study conducted on exosomes derived from *M. tuberculosis*-infected J774 cells identified 41 mycobacterial proteins, mostly predicted or known to be secreted proteins, including previously known immunogenic proteins ESAT-6, Ag85 complex proteins, MPT64, or MPT63 (61). Interestingly, some of the mycobacterial proteins in exosomes released from macrophages treated with *M. tuberculosis* CFP were identical to the ones contained in exosomes isolated from infected cells. This observation implies that mycobacterial proteins may have a signal for trafficking into exosomes upon entry into the cell *via* phagocytosis or endocytosis-based mechanisms (61). Similarly, exosomes obtained from sera of active TB patients also include *M. tuberculosis* proteins. This observation suggested that exosomes can serve as a source of peptide biomarkers for TB. In comparison to the exosomes isolated from the bronchoalveolar lavage (BAL) fluid from *M. tuberculosis*-infected BALB/c mice and infected J774 macrophages (61), five of these proteins were in common, including DnaK, PstS2, GlcB, HspX, and AcpM (62). The mycobacterial proteins contained within the host exosomes appear to be secreted *via* the SecA and type VII secretion system, although at least one of these proteins is not expected to be secreted (GabD1). This might mean that several different mechanisms, including bacterial lysis, are responsible for the release of mycobacterial proteins prior to their incorporation into exosomes (61).

The mechanisms that control the trafficking of these soluble bacterial antigens to the exosomal compartments are not currently known. For *M. tuberculosis* several pathways have been shown to be important in the antigen trafficking to exosomes. In addition to rab27a discussed above, they also showed reduced trafficking of *M. tuberculosis* 19 kDa lipoprotein, which were (59). This observation suggested the importance of rab27a pathway in the trafficking of mycobacterial proteins to exosomes.

The pathway that is crucial for the trafficking of proteins into macrophages is ESCRT dependent pathway. Knockdown of Tsg101 and Hrs, which are the ubiquitin-binding domains of ESCRT-1 and ESCRT-0, respectively, resulted in the reduction of exosomes produced in RAW 264.7 macrophages, which indicated that ubiquitin might be one of the mechanisms involved in the trafficking of proteins to MVBs and exosomes (63). Schorey et al. examined whether ubiquitination is a post-translational modification necessary for the trafficking of soluble mycobacterial antigens into the exosomes. Toward this goal, exosomes were purified from *Mycobacterium*-infected RAW264.7 macrophages, followed by pull-down of mono-ubiquitinated proteins. The western blotting of such mono-ubiquitinated proteins was done using an antibody that recognizes culture filtrate proteins of *M. tuberculosis*, and the results indicated that several mycobacterial proteins are ubiquitinated. Moreover, specific mycobacterial proteins KatG, HspX, and GroES were shown to be ubiquitinated by using this western blot technique (63). An inhibitor PYR-41 was used to treat cells prior to the exosome purification since this molecule inhibits the thioester bond formation between ubiquitin and E1. This compound treatment leads to a complete depletion of mycobacterial proteins in the collected exosomes. Further, mutation of a specific lysine residue in the mycobacterial protein HspX diminished its trafficking into the exosomes that was dependent on the clathrin-mediated endocytosis. Mono-ubiquitination is required for the trafficking of some proteins into the endosomes. Indeed, the trafficking of HspX was shown to be dependent on clathrin-mediated endocytosis, based on the treatment with Dynasore, which is an inhibitor of this uptake mechanism (63). In addition, expression of a C‐terminal fusion of ubiquitin to EGFP and *M. tuberculosis* proteins Ag85B and ESAT-6 in HEK 293 cells enhanced the delivery of these proteins into exosomes by ten-fold when they were coupled to ubiquitin (**Figure 1**). These exosomes were able to elicit a T cell response by stimulating the production of INFɣ‐secreting T lymphocytes in the lung and spleen (64).

These results collectively suggested that mono-ubiquitination could serve as a mechanism for the trafficking of bacterial proteins into the exosomes *via* clathrin-mediated endocytosis. The ubiquitin E3 ligases responsible for the ubiquitination of these bacterial proteins have not yet been identified, but it is likely that multiple E3 ligases exist that play this function in sorting of proteins to exosomes. Furthermore, apart from a better characterization of ubiquitination in this context, it would be interesting to investigate additional mechanisms that might guide mycobacterial proteins to vesicular compartments. Moreover, the fate of the mycobacterial proteins that are carried in exosomes to target cells is also unknown. It would be important to track the bacterial proteins carried to target cells *via* exosomes, in order to identify the colocalization of these proteins with intracellular compartments, such as endocytic vesicles and lysosomes. Furthermore, since exosomes can carry these mycobacterial proteins to the antigen-presenting cells, and because exosomes formed during the Mtb infection stimulate protective immunity against Mtb (50, 59), it is possible that the Mtb antigens are either loaded on MHC molecules or the entire exosomal complex containing MHC and Mtb antigenic peptide are exposed on the cell surface. The trafficking of the exosomal content within the target cell clearly deserves further mechanistic studies.

### Small Extracellular Vesicles From Mycobacterium-Infected Macrophages Carry RNA

Apart from proteins, exosomes can also transmit other molecules from infected cells. In the case of *M. tuberculosis* infection, exosomes carry a smaller amount of microRNA (miRNA) than uninfected cells. However, transcripts regulating immune response are more abundant in exosomes derived from *M. tuberculosis*-infected macrophages. Apart from the host RNA, the vesicles are also capable of carrying mycobacterial RNA. Similar to proteins, the RNA cargo is transferred between cells *via* exosomes (65). Currently, a mechanism responsible for the trafficking of bacterial transcripts to the exosomes remains uncharacterized, although RNA‐binding protein hnRNPA2B1 has been shown to bind specific miRNAs (46). This and other RNA‐binding proteins are, therefore, likely capable of sorting relevant miRNAs into the vesicles.

## Eukaryotic Pathogens

### Brief Review of *Plasmodium* biology

*Plasmodium* parasites are apicomplexan parasites that cause malaria. It is estimated that over 100 million people are infected worldwide, and just under 1 million people succumb to these infections each year (66). Infections in humans are initiated by sporozoites deposited into a host by female Anopheles mosquitoes when they take a blood meal. The sporozoites establish a productive infection when they infect liver cells. Although hepatocytes are the principal hosts of parasites in the liver, it has been shown that sporozoites take up transient residence in Kupfer cells that mediate their access to hepatocytes (67, 68). It was also shown that after infection of hepatocytes, a small subset of liver parasites is acquired by monocyte-derived CD11c+ cells (69). Infection of CD11c+ cells and hepatocytes were shown to be dependent on their expression of the tetraspanin CD81, which coincidentally is a vital component of exosomes (70). The parasitophorous vacuoles (PVs) in which the parasites reside in hepatocytes exhibit unique characteristics compared to PVs that harbor other Apicomplexan parasites. For example, *Plasmodium* containing PVs rest close to the cell nucleus and establish interactions with the Endoplasmic Reticulum (ER), which is in contrast to *Toxoplasma* whose PVs establish a tight association with the Golgi apparatus, Mitochondria, and Endoplasmic Reticulum (ER) (71) of the host cell, Furthermore, *Plasmodium*-containing PVs have been shown to have pores through which nutrients smaller than 3000 Da can be exchanged between the PV lumen and cell cytosol (71). Parasites in PVs within liver cells undergo rapid division into merozoites. The PVs enlarge into giant syncytium-like compartments (71). Upon their release from the liver, merozoites enter the blood circulation, where they infect red blood cells. Of the five species of *Plasmodium* parasites that infect humans, *Plasmodium falciparum* and *Plasmodium knowlesi* infect all red blood cells. In contrast, *Plasmodium vivax* and *Plasmodium ovale* infect only reticulocytes. *Plasmodium malariae* infect older red blood cells. Many mechanistic studies on *Plasmodium* infections have been performed in experimental models with parasites of rodents, including *P. berghei, P. yoelii* and *P. chabaudi* that exhibit similarities and differences with *Plasmodium falciparum* and *Plasmodium vivax*, depending on the stage of infection.

### Exosomes in *Plasmodium* Infections

As discussed above, during their life cycle, *Plasmodium* parasites infect distinct cell types that should be expected to employ mechanisms for exosome biogenesis. Several studies have explored the role of exosomes and microvesicles (previously called microparticles) in *Plasmodium* infections. Mature red blood cells are devoid of a nucleus and the endocytic cell machinery in their cytoplasm, including multivesicular bodies (MVBs) that play a critical role in exosome biogenesis in nucleated cells. *Plasmodium* within mature red blood cells have been shown to translocate over 300 parasite-derived proteins from their PVs into the red blood cell cytosol. These molecules that are distributed throughout the red blood cell, including on its cell surface, have been implicated in several functions, including the formation of Maurer’s clefts that appear to play some role in exosome formation in *Plasmodium*-infected cells (72). It is striking that only a subset of the molecules that are translocated into the red cell has been identified in EVs that are recovered from the supernatant fluid of cultured infected erythrocytes (73, 74). While EVs included proteins from the PV membrane and some well-studied surface molecules such as PfEBA and knob-associated molecules, they however, lacked other well studied molecules, including PfEMP1(a knob associated protein) and AMA-1. This observation suggests that there is a machinery outside of the parasite in the red blood cell that plays a pivotal role in selecting molecules for inclusion in EVs. Abdi et al. (74) analyzed exosomes released from red blood cells infected with a relatively earlier parasite passage. They identified over 50 more *Plasmodium* molecules in those EVs as compared to exosomes produced from a parasite line that had long been adapted to the *in vitro* culture conditions (74). Not surprisingly, many of the molecules that were identified in the study with the low passage parasites were involved in virulence. That study underscored the need to evaluate recently obtained field isolates and suggested that molecules in exosomes may play a role in the parasites’ virulence.

A couple of elegant studies have shown that exosomes can mediate intracellular communication between parasites within infected red blood cells [reviewed in (75)]. Studies using transgenic parasites expressing a drug resistance marker showed that DNA packaged in EVs could be exchanged by parasites in an infected cell, which results in the spread of a resistance marker (72). Another study showed that infected red blood cells selectively took up EVs produced by other infected cells, which then stimulated gametocyte production in the recipient infected cells (73). Exosomes have also been shown to stimulate the immune response by activating macrophages and neutrophils [reviewed in (75)]. They also play significant roles in cerebral malaria where among other activities, they promote vascular changes, including endothelial cell activation [reviewed in (76)]. Together, those studies demonstrated the critical role of EVs in *Plasmodium* infections and suggested that a greater understanding of their biogenesis and functions could be exploited to modify the course of malaria.

Studies on the ATP binding cassette transporter A1 have suggested a role for this molecule in EV biogenesis in *Plasmodium* infections. It had been shown that ATP binding cassette transporter A-1 (ABCA-1) plays a role in phosphatidylserine distribution at the plasma membrane (77). ABCA-1 knock-out mice were subsequently shown to be defective in EV release. Red blood cells from these mice produced reduced levels of EVs, which implicated this molecule in EV biogenesis in red blood cells [reviewed in (76)]. As mentioned briefly above, the molecules localized to Maurer’s clefts were identified in EVs from *Plasmodium*-infected RBCs. Studies by Regev-Rudzki et al. (72) identified and tracked the PfEMP1 trafficking protein (PfPTP) that associates with Maurer’s cleft (72). They proceeded to show that parasites that were genetically altered to lack expression of PTP-2 were defective in EV release when used to infect red blood cells. This provided compelling evidence of the role of Maurer’s clefts in EV biogenesis in infected cells.

In contrast to mature red blood cells, reticulocytes are nucleated and possess the biosynthetic machinery of mammalian nucleated cells. The composition and functions of exosomes released from *Plasmodium*-infected reticulocytes and liver cells have been described. The studies of Martin-Jaular et al. (78) that characterized exosomes released by BALB/c mice infected with non-lethal *Plasmodium yoelii* 17X described the presence of parasite-derived molecules in exosomes from infected reticulocytes (78). In that experimental model, where *Plasmodium yoelii* 17X infects reticulocytes, infections were initiated by intraperitoneal injection of infected cell blood cells. Up to 31 parasite-derived proteins were found to be included among the molecules in the reticulocyte-derive exosomes. Some of the parasite proteins that were identified included the serine-rich antigen (SERA) that is expressed by a multigene family and has been implicated in virulence [reviewed in (79)], merozoite surface antigens (MSP1 and MSP9), and heat shock protein 70. The mechanisms that led to their inclusion in reticulocyte derived exosomes have not been described. A more recent study by Gualdrón-López et al. (80) described the characterization of exosomes that were secreted from the liver stages of *Plasmodium vivax* infections (80). The research team took advantage of the human liver-chimeric (FRG huHep) mouse (81) in which an immunocompromised mouse with several genetic mutations was engrafted with human hepatocytes. FRG huHep mice support the complete development of the human parasites, *P. falciparum* (81) and *P. vivax* (82) that would not otherwise infect mice. The analysis of exosomes isolated from the blood plasma of the *P. vivax* infected FRG huHep mice (ExEF) identified 290 and 234 proteins from mouse and human origin, which included liver proteins that had previously been described from liver exosomes (80). This analysis also identified 17 parasite-derived molecules that were included in exosomes from liver-infected cells. When the authors compared human ExEF exosomes from infected animals versus uninfected control mice, they stated that several proteins were differentially associated with *P. vivax* infections (*P*-value < 0.05). This finding is consistent with studies from other infections that have shown that the infection influences host-derived molecules’ composition in exosomes. The list of parasite proteins in ExEF included heat shock protein 70 (HSP70), which was also seen in the study on infected reticulocyte-derived exosomes described above. A different variant of merozoite surface antigen (MSP3) was found in the ExEF, which differed from the variants found in the infected reticulocyte derived exosomes discussed above. Whether it is 17 or 31 parasite-derived proteins identified in exosomes from infected liver cells or from infected reticulocytes, it is currently unknown which characteristics of these proteins direct them to the host cellular exosome biogenesis machinery. It is also not known whether ubiquitination plays a role in exosome loading within *Plasmodium-*infected cells.

### Toxoplasma

*Toxoplasma* are Apicomplexan parasites that can infect all warm-blooded animals, including mammals and birds. In humans, *Toxoplasma* have been implicated in a range of clinical presentations whose severity is determined by the individual’s immune status. *Toxoplasma* is acquired by ingestion of raw or inadequately cooked meat. It can also be acquired upon ingestion of oocyst dispersed in the environment in cat feces. This later mode of acquiring the infection by pregnant women is the subject of public health campaigns that dissuade women from changing cat litter, as infection during pregnancy can lead to congenital transmission resulting in stillbirths or hydrocephalus or retinal infections of the newborn. *Toxoplasma* can infect all nucleated cells in mammals. Parasite entry into cells involves the sequential deployment of molecules from Apicomplexan-specific organelles. The release of proteins from Rhoptries follows the discharge of micronemal proteins. Dense granule proteins are then released, which contribute to the formation of the parasitophorous vacuole and the intravacuolar network. Rastogi et al. (83), Håkansson (84), and Nadipuram (85) have described the export machinery of proteins from the *Toxoplasma* PVM and provided examples of molecules that are transported to the host cell (83–85). Whether displayed on the PVM or translocated to other host cell organelles or the cytosol, most of these molecules are potentially accessible to the exosome biogenesis machinery.

Studies of the potential role of exosomes in toxoplasmosis have evaluated exosomes that are released from axenic cultures of parasites (86) or exosomes that are released from dendritic cells that are pulse with Toxoplasma antigens (lysate) (87, 88) or mammalian cells that are infected with Toxoplasma (56, 89). Despite the obvious differences in the sources of *Toxoplasma* molecules, exosomes containing *Toxoplasma* molecules were shown to be able to stimulate naïve recipient cells to secrete cytokines. Injection into hosts in experimental studies led to the elaboration of a variety of *Toxoplasma* specific responses. Some studies of exosomes secreted from *Toxoplasma*, have shown that infection with *Toxoplasma* induces the release of a unique profile of protein and nucleic acids that is different from those released by uninfected cells. In infections of human foreskin fibroblast cells, Wowk analyzed exosomes from cells infected with *Toxoplasma gondii* and compared their protein content to exosomes from axenic cultured tachyzoites. They found 69 unique parasite derived proteins in infection derived EVs, however the number of parasite derived proteins was not stated (90). In another study in which dendritic cells were infected with *Toxoplasma gondii*, 12 differentially expressed miRNAs compared to exosomes from uninfected cells were identified (91). Further analysis predicted that in recipient cells, these miRNAs could be associated with a variety of biological processes, including signaling pathways involved in host ubiquitin system, innate immunity, biosynthesis, and transferase activity. Future studies will no doubt provide greater insight on the trafficking of these parasites derived molecules in the infected host cells.

### Leishmania

#### Brief Review of *Leishmania* Infections

*Leishmania* are members of the family Trypanomastidae in the order Kinetoplastida. *Leishmania* parasites are grouped into two subgenera: *Leishmania* (*Leishmania*) and *Leishmania* (Viannia) that are further classified into species and subspecies. Infection of humans results in a disease presentation that is mostly dependent on the parasite species. Parasites of the *Leishmania* (*Leishmania*) subgenus including *L.(L) donovani*, *L.(L) infantum* and *L.(L) chagasi* (with a few exceptions including *L.(L) amazonensis and L.(L) major, L.(L) tropica* that cause cutaneous infections) are the primary causative agents of visceral disease. In contrast, parasites of the *Leishmania* (Viannia) subgenus, including *L.(V) braziliensis*, *L.(V) panamensis*, cause cutaneous infections that can manifest as self-limiting lesions or disseminated lesions or mucocutaneous infections [reviewed in (92, 93)]. However, there are reports of visceral disease caused by parasites that ordinarily lead to cutaneous lesions, while other reports of cutaneous lesions have implicated parasites species that typically lead to visceral disease [reviewed in (94)]. These ‘unexpected’ disease presentations underscore the complexity of these infections and may be due to host genetics contributions and poorly defined environmental factors (94, 95). *Leishmania* parasites are transmitted by sandflies. Once inside the mammalian host, *Leishmania* infects phagocytic cells wherein they reside in *Leishmania*-containing parasitophorous vacuoles (LPVs). The *Leishmania* species determine LPV morphology. At the extremes of morphological differences, parasites of the *L. mexicana* complex (*L. mexicana*, *L. amazonensis*) reside within large communal LPVs that continuously distend. At the other extreme, *L. donovani*, *L. chagasi/L. Infantum* reside in tight LPVs that harbor a single parasite. After parasite replication and fission, daughter parasites segregate into secondary LPVs that also house individual parasites. All other *Leishmania* species reside in LPVs that may house one to four parasites. It is presently not known how LPV morphology differences affect *Leishmania* pathogenesis. LPVs are dynamic compartments composed of molecules from the host secretory pathway, including the endoplasmic reticulum (ER) and the endocytic pathway, including late endosomes and lysosomes (96). Although much has been learned about the molecular composition of LPVs, there are still unanswered questions about the biology of LPVs, including how *Leishmania* derived molecules are translocated across the LPV membrane and which signals mediate the trafficking of parasite-derived molecules to the intracellular sites where they express their functions. On this topic, a recent publication provided evidence of traffic of *Leishmania* proteins from LPVs to the ER in vesicles that otherwise transmit cargo between the Golgi and the ER (97). The authors proposed that this could be one route through which parasite molecules are retrieved from LPVs for distribution in the infected cell and beyond.

In natural infections, the parasite’s promastigote form is deposited in the skin of the host by the sandfly. The parasites commence a skin phase of the infection. After a transient residence in neutrophils, parasites are transferred to macrophages wherein they undergo replication as amastigotes (98, 99). In cutaneous infections, inflammatory cells are recruited to the bite site, which results in a cutaneous lesion over time. In infections by parasites that cause visceral infections, there is also an initial cutaneous phase (can last for several weeks) after which infected cells migrate to visceral organs where they replicate and form inflammatory lesions; this was first demonstrated in infections of hamsters (100). Parasites such as *L. amazonensis* and *L. braziliensis* that disseminate to secondary sites, proliferate for much longer periods at the primary site (months to years) before dissemination. Recent studies that have explored the phenotype of cells at primary and secondary sites in experimental infections with *L. major*, highlighted the expression of chemokine receptors CCR2^+^ and CX3CR1^+^ on the monocyte-derived cells that are the primary host cells of these parasites (99, 101). Infections in knockout mice that lack these receptors were limited, which suggested a role for chemokines in *Leishmania*-infected cell dissemination [Also reviewed in (102)]. Nonetheless, neither the triggers for disseminating the infection nor the factors that determine the selection of secondary sites to which parasites spread are not known. Could some of the mechanisms that promote metastatic tumor dissemination, such as the roles played by exosomes in tissue homing or organotropism (reviewed in (103, 104), be also important in the dissemination of *Leishmania* infections?

After parasites take up residence at a tissue site, remodeling of the site ensues. Analyses of skin lesions in humans revealed the noticeable presence of blood vessels of varying morphologies at these sites (105, 106). Experimental infections of cutaneous and visceral leishmaniasis also undergo vascular changes at the lesion site. Horst et al. (107) reported that cutaneous infection by *L. major* parasites in the hindfoot of C57BL/6 wild-type mice resulted in extensively vascularized lesions because the lymphatic and blood vessels were readily evident as the infection established (107). The authors implicated the expression of carcinoembryonic antigen-related cell adhesion molecule 1 (CEACAM1) on mononuclear cells (CD11b^hi^ cells) as essential mediators of angiogenesis in *L. major* infected lesions. Also performing studies with *L. major*, Weinkopff et al. (108), showed that there was increased expression of vascular endothelial growth factor-A (VEGF-A) and vascular endothelial growth factor receptor (VEGFR-2) at the site of infection that mirrored the increase in lesion size and parasite numbers (108). In infections with *L. donovani*, where lesions form in visceral tissue, Yurdakul et al. (109), described vascularization and neovascularization of the red pulp and white pulp regions of the spleen, respectively (109). They attributed splenic vascularization to Ly6C+ inflammatory monocytes. In a more recent study, Dalton et al. (110), showed that neurotrophic tyrosine kinase receptor type 2 (Ntrk2, also known as TrkB) was aberrantly expressed on splenic endothelial cells following *Leishmania* infections (110). The study then showed that macrophages expressed the ligand(s) for Ntrk2 in the infected spleen and that inhibition of signaling through Ntrk2 blocked white pulp neovascularization.

#### Exosomes in *Leishmania* Infections

Natural *Leishmania* infections are initiated with promastigotes, which are a transient stage within the mammalian host. Within 24 hours of internalization into macrophages, they transform into amastigotes. The characteristics of this transformation that is triggered by temperature and pH changes were described by Zilberstein and colleagues (111, 112). The present challenge is to determine which molecules are released from long-term infections. Many *Leishmania* virulence factors have been described; however, as Kaye et al. (113) noted in their recent review, no *Leishmania* parasite-derived factors that cause tissue damage are known (113). It is likely, though, that exosomes released from infected cells carry molecules that could play a role in lesion development and immune response activation. *Leishmania* parasites themselves produce a variety of extracellular vesicles, including exosomes, which enable them to interact with and respond to their environment. *Leishmania* promastigotes were found to release vesicles with an average diameter of 30-70nm, consistent with exosomes released by other cell types (114). The molecular composition of these parasite-derived exosomes has been evaluated and shown to contain homologs of some mammalian exosome markers, as well as molecules that may enhance infectivity (114, 115). Up to 329 molecules have been identified in exosomes released from axenic promastigotes, accounting for greater than 52% of the parasite secretome (115). Atayde et al. (116) showed that *L. major* parasites secrete exosomes within the sandfly midgut, which, when injected with parasites during the initial insect bite of a mammalian host, enhance infection and lesion development in mice (116). Additionally, the exosomes produced by axenic *Leishmania* promastigotes modulate the chemotactic activity and cytokine secretion of macrophages *in vitro* to suppress the immune response and enhance permissiveness to subsequent infection (115). The vesicles also have the capacity to activate the immune system of the host. Exosomes released from *L. major* parasites were shown to induce Th2 polarization and enhanced disease progression in mice, indicating that parasite-derived vesicles are immunosuppressive and proparasitic in nature.

The *Leishmania* major surface protease (MSP), also referred to as GP63, is an important virulence factor, which contributes to enhanced phagocytosis by macrophages and promoting the survival of the parasite during both promastigote and amastigote life stages. There are three pools of GP63: surface-localized, internal, or released, that are believed to traffic separately through the cell and then released into the extracellular environment (117). Upon infection of macrophages, released GP63 is captured in vesicles and may also access the macrophage cytoplasm, though the exact mechanism of GP63 delivery to the host cytoplasm remains unclear; *Leishmania* parasites lack secretion systems comparable to those found in pathogenic bacteria (118). Exosomes released by *L. mexicana*-infected J774 macrophages for 24 hours were found to contain GP63, suggesting that parasite molecules from this intracellular pathogen can access host exosomes and then be released widely (119).

Recent research also indicates that macrophages infected with *L. donovani* amastigotes release exosomes containing a mixture of unique host and parasite proteins contributing to pathogenic processes (120). In that study, infections of RAW264.7 macrophages were initiated with *L. donovani* promastigotes, and infections were allowed to continue for 72 hours (about 3 days) to evaluate mature infections. After 72 hours of infection, extracellular vesicles were purified from media supernatants using differential centrifugation and then subjected to liquid chromatography combined with tandem mass spectrometry. In-solution and in-gel protocols for tryptic digestion were used prior to the mass spectrometry-based analysis, and data combined to yield protein composition profiles for uninfected and infected macrophage-derived EVs. Consequently, this approach led to a confident identification of 59 parasite proteins in EVs released by infected macrophages. These proteins included a putative Vasohibin, nucleoside transporter 1, kinesin, DNA directed RNA polymerase II subunit 2, dynein heavy chain, and putative protein kinases. Interestingly, some of the same protein families were also identified in a study of circulating immune complexes in the peripheral blood of 115 human patients with active *L. donovani* infections (121). In that clinical study, circulating immune complexes (CICs) were purified from patient serum using PEG-assisted precipitation and centrifugation, CIC antigens and antibodies were then dissociated using an acidic buffer, and the antibody was removed using protein A agarose to yield purified antigens. The researchers then used 2D gel electrophoresis and mass spectrometry of these purified CIC antigens to identify parasite proteins, revealing 31 proteins present during active infection before drug treatment. While there appears to be an elevated level of congruence between the parasite-derived protein profiles of these CICs and EVs isolated from infected macrophages, it is difficult to resolve this relationship past the family level. Finding similarities when comparing data between projects can be complicated due to differences in mass spectrometry approach, poor protein annotations, and the overall redundancy of *Leishmania* protein databases currently in use. In addition, it can be challenging to match proteins exactly across different experiments by reported accession numbers. However, the parity between these profiles at the protein family level remains intriguing. Along with what is known about exosomes and their ability to circulate through the body and deliver their contents to tissues distant from their origin, these findings suggest that *L. donovani* infected macrophages release exosomes containing parasite factors that may aid in their circulation throughout the host.

It is also worth noting that exosomes released by infected macrophages contain only 59 parasite-derived proteins, a significantly smaller number than the 329 parasite proteins identified in *Leishmania* promastigote secreted exosomes (122). Comparing the parasite-derived profiles of exosomes from promastigotes versus exosomes from amastigote infected macrophages, we again see several overlapping protein families, including elongation factor 1-alpha, serine/threonine-protein kinase, kinesin, and calpain-like cysteine peptidase. Strikingly, many of the molecules present only in promastigote-derived exosomes are homologs of mammalian exosome structural components (5). The absence of these parasite proteins in exosomes released by infected macrophages may be the result of two biological phenomena. First, the expression of parasite proteins may be differentially regulated between the promastigote and amastigote life stages. For example, a sizable portion of promastigote proteins are downregulated during macrophage infection as the parasites adapt to life in the intracellular environment. Alternatively, it may be unnecessary for the structural proteins of EVs to be contributed by the parasites during infection, as the parasites may be able to hijack the existing host machinery for protein secretion and exosome biogenesis. To be sure, the mammalian counterparts of these structural proteins were identified in EVs from infected macrophages, suggesting that *Leishmania* amastigotes may translocate their molecules across the LPVM into the host cytosol to utilize normal host processes to their advantage for the secretion of specific parasite molecules. EVs released by *Leishmania*-infected macrophages were also found to enhance several measures of endothelial cell activation *in vitro*, including tube formation, cell migration, and enhanced production of VEGF and IL8, which suggests that infection-induced EVs may play a role in neovascularization and pathogenesis (120).

## Conclusion

All eukaryotic cells release EVs, including exosomes. The biogenesis of EVs released by cells under various normal and abnormal conditions has been well-studied. There is ample evidence that intrinsic cell characteristics and environmental queues determine the composition of EVs. This results in unique exosome compositions that could be monitored for diagnostic value. Ongoing studies have shown that intracellular pathogens, including viruses, bacteria, and parasites, can take advantage of the host exosome machinery to release virulence factors. Pathogens may smuggle pathogen-encoded virulence factors and other molecules that collectively contribute to their pathogenesis. The existence of these mechanisms suggests that pathogens have evolved adaptations to take advantage of host protein trafficking mechanisms for exosomal packaging. For its part, the host cells also modulate exosome composition as an anti-pathogen strategy since exosomes induce immune responses directed against specific pathogens. More research is needed to evaluate specific mechanisms by which pathogen-derived molecules are targeted to multivesicular bodies within infected cells and further packaged into host-derived exosomes. This information will elucidate a large gap in our understanding of intracellular host-pathogen interactions and identify novel drug targets for infection control.

## Author Contributions

AG wrote and edited the manuscript. ME wrote and edited the manuscript. PEK developed the manuscript focus, wrote and edited manuscript. All authors contributed to the article and approved the submitted version.

## Funding

PEK was supported by an R56 AI143293 grant from the NIH. ME was supported by the R03 AI-135610 grant from NIH.

## Conflict of Interest

The authors declare that the research was conducted in the absence of any commercial or financial relationships that could be construed as a potential conflict of interest.
